# Anti-Photoaging Effect of *Phaseolus angularis* L. Extract on UVB-Exposed HaCaT Keratinocytes and Possibilities as Cosmetic Materials

**DOI:** 10.3390/molecules28031407

**Published:** 2023-02-01

**Authors:** Sarang Oh, Shengdao Zheng, Minzhe Fang, Myeongju Kim, Arce Defeo Bellere, Jeehaeng Jeong, Tae-Hoo Yi

**Affiliations:** 1 Snowwhitefactory Co., Ltd., 807 Nonhyeon-ro, Gangnam-gu, Seoul 06032, Republic of Korea; 2 Graduate School of Biotechnology, Kyung Hee University, 1732 Deogyeong-daero, Giheung-gu, Yongin-si 17104, Republic of Korea

**Keywords:** *Phaseolus angularis* seed extract (PASE), photoaging, cosmetic material, UV (ultraviolet)B

## Abstract

*Phaseolus angularis* L. is widely cultivated and is considered a superfood because of its nutritious protein and starch contents. Nevertheless, *P. angularis*’s effects on skin photoaging are unknown. The aim of this study was to research the effects of *P. angularis* seed extract (PASE) on photoaging in human keratinocytes (HaCaT) damaged by UVB radiation so as to find out whether PASE can be used as an effective anti-photoaging ingredient in cosmetic products. The antioxidant activities were assessed using 1,1-diphenyl-2-picrylhydrazyl (DPPH), 2,2′-azino-bis-(3-ethylbenzothiazoline)-6-sulfonic acid (ABTS) radical scavenging, and reactive oxygen species (ROS) assays. Enzyme-linked immunosorbent assay (ELISA) analysis was used to determine the change in matrix metalloproteinase (MMP)-1, and MMP-3. The protein levels of mitogen-activated protein kinase (MAPK)/activator protein (AP)-1, transforming growth factor beta (TGF)-β/suppressor of mothers against decapentaplegic (Smad), and NF-E2-related factor (Nrf)2/antioxidant response element (ARE) were measured by western blot. As a result, PASE increased DPPH and ABTS antioxidant activities in a dose-dependent manner. Additionally, PASE treatment (100 µg/mL) significantly reverted the damage induced by UVB (125 mJ/cm^2^) irradiation by downregulating ROS, matrix metalloproteinase (MMP)-1, and MMP-3 secretion and expression and increasing procollagen type I production. To suppress MMP-1 and MMP-3 secretion, PASE significantly decreased UVB-induced p38 and JNK phosphorylation and phosphorylated c-Fos and c-Jun nuclear translocation. PASE promoted collagen I production by inhibiting UVB-induced TGF-β activation and Smad7 overexpression; antioxidant properties also arose from the stimulation of the Nrf2-dependent expression of the antioxidant enzymes heme oxygenase (HO)-1 and quinone oxidoreductase (NQO)-1. Our data demonstrated that PASE has the potential to prevent ROS formation induced by UVB exposure by targeting specific pathways. Thus, PASE might be a potent anti-photoaging component to exploit in developing anti-aging products.

## 1. Introduction

As age progresses in the skin, functional features and normal structure decrease. This is caused by endogenous aging and extrinsic aging, which are clearly distinguished biologically. The most important of these factors is solar ultraviolet (UV) radiation, and skin aging caused by UV rays is called photoaging [1]. UVA and UVB activate signaling pathways within cytoplasm associated with temporary and permanent gene damage and differentiation, growth, aging, and associated tissue regression [2]. Ultraviolet rays affect the intracellular signaling system of the skin, causing DNA and gene expression damage, resulting in cell aging and cell apoptosis [3].

Exposing the body to UV rays induces the generation of reactive oxygen species (ROS) in cells. These ROS in excess destroy collagen, elastin, and the extracellular matrix. They also promote the expression of proteolytic enzymes and matrix metalloproteinases (MMPs), leading to the breakdown of protein fibers. Thus, excessive ROS production participates in the changes in skin collagen implicated in the skin aging process and reduces skin wrinkles and elasticity [4].

Moreover, ROS activates the nuclear factor-kappa B (NF-κB) pathway, which induces the expression of inflammatory cytokines, and other intracellular signaling pathways through mitogen-activated protein kinase (MAPK), p38, and c-Jun N-terminal kinase (JNK) [5]. These enzymes are released from the cell membrane and induce the expression of the nuclear transcription complex activator protein-1 (AP-1) [6]. AP-1 decreases the expression of the procollagen 1 gene and transforming growth factor beta (TGF-β) receptor levels by increasing the transcription of MMPs. The decrease in TGF-β receptor expression inhibits the synthesis of procollagen and promotes the production of the proteolytic enzyme MMP-1, consequently increasing the rate of collagen degradation and causing the dermal matrix layer to collapse and photoaging to occur. Thus, it is important to reduce ROS levels to suppress photoaging [7].

When the skin is exposed to UV light and ROS are produced, NF-E2-related factor 2 (Nrf2) is activated and binds to the antioxidant response element (ARE) [8]. The activation of the Nrf2/ARE pathway induces the expression of antioxidant-related genes, such as genes coding for NAD(P)H quinone oxidoreductase 1 (NQO1), heme oxygenase-1 (HO-1), and dihydrolipoic acid (DLD), thereby reducing cellular oxidative stress [9]. However, with aging, the activity of the Nrf2-dependent pathways decreases, leading to a lower expression of antioxidant enzymes. Therefore, the activation of the Nrf2 pathway is a very important antioxidant defense mechanism for preventing skin aging induced by ROS.

*Phaseolus angularis* seeds have various effects, such as alleviating edema, eliminating inflammation, and relieving poisoning effects [10]. Moreover, they are rich in vitamin B1, which helps to recover from fatigue and is effective against symptoms, such as digestion difficulties, absorption impairment, and memory loss [11]. *P. angularis* has been reported to exert antidiabetic, antioxidant, and anti-inflammatory effects [12,13,14]. *P. angularis* seed coats, which are rich in polyphenols, have recently been shown to inhibit melanin biosynthesis and attenuate vascular oxidative stress in spontaneously hypertensive rats [15,16]. Nevertheless, there are no reports regarding the anti-photoaging properties of *P. angularis* seeds

The present study was conducted to contribute to the development of natural photoaging inhibitors by evaluating the anti-photoaging effects of *P. angularis* seed extract (PASE) and its inhibitory effects on aging mediators in human keratinocytes (HaCaT cells) exposed to UVB. The anti-photoaging effects of PASE were investigated by analyzing the levels of ROS, MMPs, and procollagen type I as well as MAPK/AP-1, TGF-1/suppressor of mothers against decapentaplegic (Smad), and Nrf2/ARE signaling.

## 2. Results

### 2.1. Analysis of Chemical Contents of PASE

**Figure 1:**
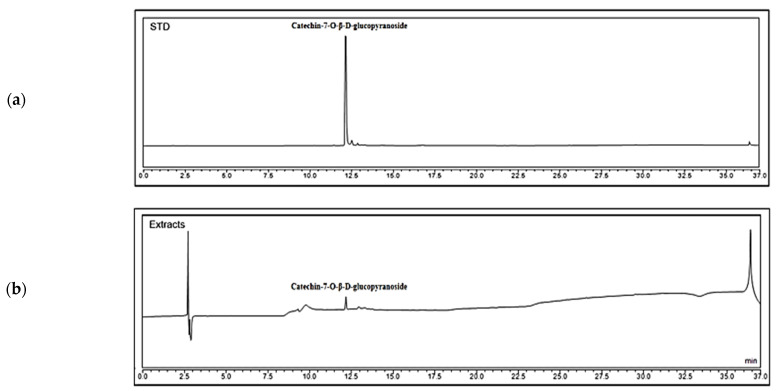
HPLC analysis of PASE: HPLC analysis of catechin-7-O-β-D-glucopyranoside standards (**a**) and the catechin-7-O-β-D-glucopyranoside content of PASE (**b**).

To prepare PASE samples, we extracted dried *P. angularis* seed powder (100 g) with 70% ethyl alcohol and obtained 10.44 g of PASE crude product. As shown in Figure 1, catechin-7-O-β-D-glucopyranoside was identified in PASE at 12 min by comparing with the retention times obtained using the standards. The content of catechin-7-O-β-D-glucopyranoside in PASE was 2.2%.

### 2.2. Total Phenolic and Flavonoid Contents of PASE

Phenolic compounds are important plant constituents with redox properties responsible for antioxidant activity [17]. Moreover, flavonoids are secondary metabolites with antioxidant activity, the potency of which depends on the number and positions of free OH groups. PASE contained high concentrations of total phenols (144.69 ± 7.30 mg gallic acid/g dry extract) and flavonoids (133.90 ± 0.01 mg quercetin/g dry extract).

### 2.3. Antioxidative Activity of PASE

**Figure 2:**
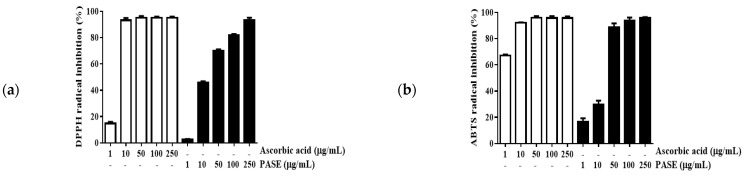
DPPH and ABTS radical scavenging activity of PASE: DPPH radical (**a**) and ABTS+ cation (**b**) scavenging activity of PASE. Ascorbic acid was used as a positive control. Data are presented as means ± SDs of three replicates.

To estimate the antioxidative properties of PASE, its radical scavenging activity was evaluated using 1,1-diphenyl-2-picrylhydrazyl (DPPH) and 2,2′-azino-bis-(3-ethylbenzothiazoline)-6-sulfonic acid (ABTS) assays. Ascorbic acid is a well-known antioxidant compound used as the reference standard and has very strong DPPH and ABTS radical scavenging activities. Here, ascorbic acid and PASE inhibited the formation of DPPH and ABTS radicals in a concentration-dependent manner. As shown in Figure 2, the IC_50_ values of ascorbic acid required for exerting its scavenging activity on DPPH and ABTS radicals were 2.5 and 0.3 μg/mL, respectively. PASE also significantly inhibited DPPH radicals, with an IC_50_ value of 15.5 μg/mL, and ABTS radicals, with an IC_50_ value of 16.1 μg/mL, suggesting that PASE possesses potent antioxidant properties.

### 2.4. Toxicity and Cytoprotective Effects of PASE

**Figure 3:**
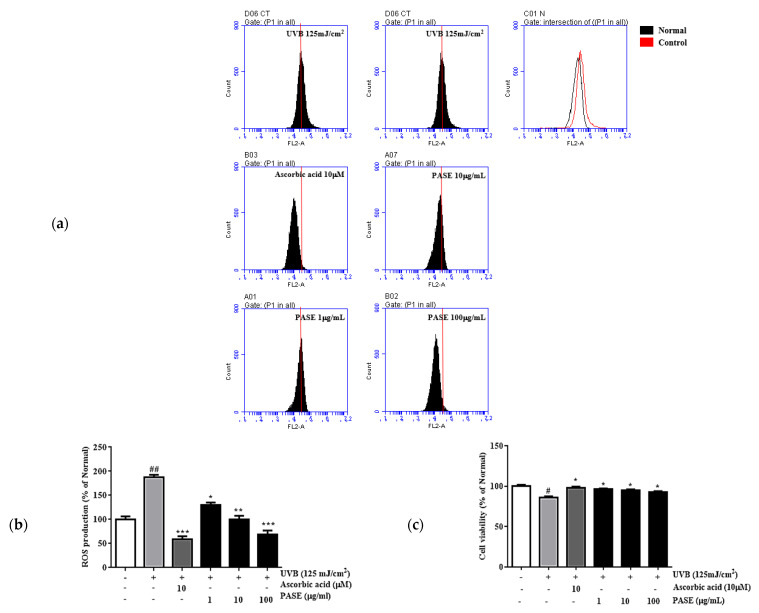
Cell viability and intracellular ROS levels in UVB-exposed HaCaT cells treated with PASE: Intracellular ROS levels are plotted against DCFH-DA levels detected by the FL-2 channel (**a**). The relative intensity is shown in the histogram (**b**). Cell viability was measured using MTT assays (**c**). Data are shown as means ± SDs of three independent experiments. # *p* < 0.05, ## *p* < 0.01 compared with non-exposed group, * *p* < 0.05, ** *p* < 0.01, *** *p* < 0.001 compared with only UVB-exposed group.

The protective effects of PASE (1, 10, and 100 µg/mL) on UVB-exposed cells were examined. As shown in Figure 3c, the cell viability was greater than 90% for all PASE concentrations, confirming that none of the tested concentrations of PASE was cytotoxic. Therefore, PASE concentrations ranging from 1 to 100 µg/mL were used for further experiments. As shown in Figure 3a, UVB significantly induced ROS generation, whereas ROS production was reduced significantly in UVB-exposed cells treated with PASE. Indeed, compared with ROS levels in UVB-exposed cells, treatment with 10 and 100 µg/mL PASE reduced ROS production by 46.6% and 63.2%, respectively. Ascorbic acid, used as positive control, inhibited ROS levels by 68.5%.

### 2.5. Effect of PASE on Collagenase and Elastase Inhibition in UVB-Exposed HaCaT Cells

**Figure 4:**
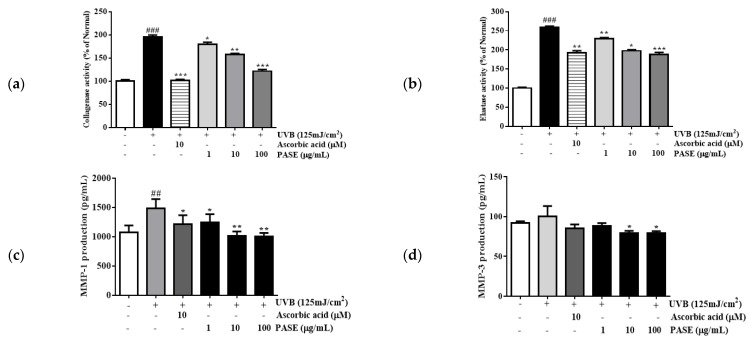
PASE inhibited the secretion of collagenase, elastase, MMP-1, and MMP-3 in UVB-exposed HaCaT cells: Collagenase (**a**), elastase (**b**), MMP-1 (**c**), and MMP-3 (**d**) secretion by UVB-exposed HaCaT cells. Data are shown as means ± SDs of three independent experiments. ## *p* < 0.01, ### *p* < 0.001 compared with non-exposed group, * *p* < 0.05, ** *p* < 0.01, *** *p* < 0.001 compared with only UVB-exposed group.

To assess the inhibitory effect of PASE on collagenase and elastase activity, we quantified the protein levels of collagenase and elastase secreted by UVB-exposed cells in the culture medium. Compared with the levels secreted by the nontreated group, UVB irradiation induced a 195.8% and 259.3% augmentation of secreted collagenase and elastase protein levels, respectively. A total of 100 μg/mL PASE treatment reduced the release of collagenase and elastase by 37.9% and 27.7%, respectively, compared what that in the UVB-exposed control group (Figure 4a,b). Ascorbic acid inhibited collagenase and elastase activation by 48.4% and 25.9%, respectively.

### 2.6. Effect of PASE on MMP-1 and MMP-3 Secretion in UVB-Exposed HaCaT Cells

To observe the inhibitory effect of PASE on collagen degradation, we quantified the protein levels of MMP-1 and MMP-3 secreted by UVB-exposed cells in the culture medium using ELISA. Compared with the levels secreted by the nontreated group, UVB irradiation induced a 38.4% and 8.9% augmentation of secreted MMP-1 and MMP-3 protein levels, respectively. PASE reversed this effect in a dose-dependent manner. Indeed, the release of MMP-1 and MMP-3 was inhibited by 32.7% and 21.9%, respectively, compared with that in the UVB-exposed control group (Figure 4c,d). Ascorbic acid inhibited MMP-1 and MMP-3 production by 18.5% and 15.4%, respectively.

### 2.7. Effect of PASE on MMP-1, TGF-β1, and Procollagen Type I mRNA Expression in UVB-Exposed HaCaT Cells

**Figure 5:**
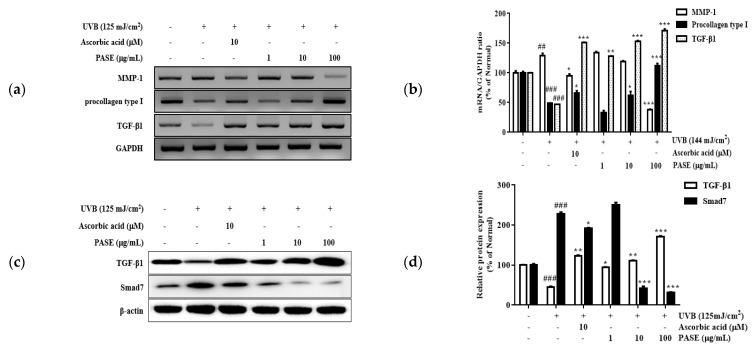
PASE inhibited MMP-1, procollagen type I, and TGF-β1 mRNA expression and TGF-β1 and Smad7 protein levels in UVB-exposed HaCaT cells: MMP-1, procollagen type I, and TGF-β mRNA levels (**a**); TGF-β1/Smad7 protein expression (**c**); the band intensities (**b**,**d**). Data are presented as means ± SDs of three independent experiments. ## *p* < 0.01, ### *p* < 0.001 compared with non-exposed group, * *p* < 0.05, ** *p* < 0.01, *** *p* < 0.001 compared with only UVB-exposed group.

Increased MMPs levels and decreased procollagen and TGF-β amounts are a hallmark of photoaging [18]. To verify the effects of PASE on MMP-1, procollagen type I, and TGF-β1 in UVB-exposed HaCaT cells, mRNA levels were measured using RT-PCR (Figure 5a,b). The expression of MMP-1 was significantly increased by UVB irradiation. Treatment with 100 μg/mL PASE effectively decreased the expression of MMP-1 by 70.1% compared with that in UVB-exposed cells. Moreover, UVB irradiation downregulated procollagen type I and TGF-β1 mRNA levels. In contrast, PASE (100 μg/mL) increased procollagen type I and TGF-β1 mRNA levels by 129.5% and 268.4%, respectively, compared to those in non-exposed HaCaT cells.

### 2.8. Effect of PASE on TGF-β1/Smad7 Activation in UVB-Exposed HaCaT Cells

TGF-β1 activation plays an essential role in collagen synthesis. Smad7 inhibits the activation of TGF-β1 and, consequently, collagen synthesis [19]. To determine the effects of PASE on TGF-β1 and Smad7 in UVB-exposed HaCaT cells, TGF-β1 and Smad7 levels were measured using a western blot (Figure 5c,d). The expression of TGF-β1 was significantly decreased by UVB irradiation. However, 100 μg/mL PASE effectively increased TGF-β1 expression by 286.1% compared with that in UVB-exposed cells. UVB irradiation also upregulated Smad7 expression, whereas PASE treatment (100 μg/mL) induced an 86.2% decrease in Smad7 levels compared with those in UVB-exposed HaCaT cells.

### 2.9. Effect of PASE on MAPK/AP-1 Phosphorylation in UVB-Exposed HaCaT Cells

**Figure 6:**
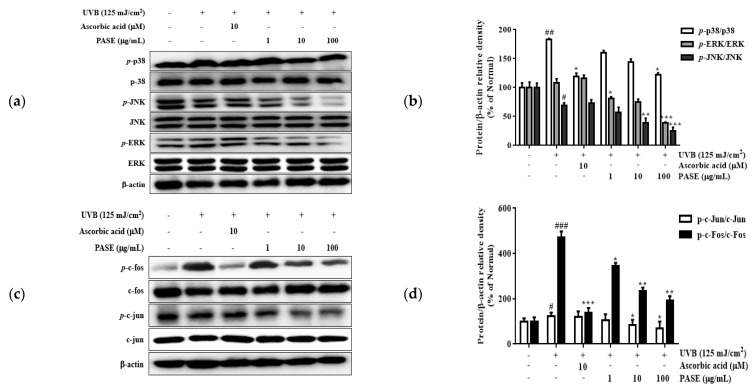
PASE inhibited the phosphorylation of MAPK/AP-1-signaling protein levels in UVB-exposed HaCaT cells: Phosphorylated and non-phosphorylated members of the MAPKs (ERK, JNK, and p38) (**a**) AP-1 complex (c-fos and c-jun) (**c**) protein levels; the band intensities (**b**,**d**). Data are shown as means ± SDs of three independent experiments. # *p* < 0.05, ## *p* < 0.01, ### *p* < 0.001 compared with non-exposed group, * *p* < 0.05, ** *p* < 0.01, *** *p* < 0.001 compared with only UVB-exposed group.

The phosphorylation of MAPK leads to the activation of the transcription factor c-jun, which can be found in the nucleus. Activated c-jun assembles with c-fos to form the AP-1 transcription factor complex. AP-1 binding sites are located in the MMP-1 promoter area, and AP-1 is an important transcription activator of MMP-1. To further investigate the effects of PASE on MAPK/AP-1 signaling in UVB-exposed cells, the expression of phosphorylated ERK (p-ERK), JNK (p-JNK), p38 (p-p38), c-fos (p-c-fos), and c-jun (p-c-jun) was measured using western blot (Figure 6). We found a significant increase in p-ERK, p-JNK, p-p38, p-c-fos, and p-c-jun levels in HaCaT cells after UVB irradiation. PASE diminished the activation of the MAPK/AP-1 pathway in a dose-dependent manner. The levels of p-ERK, p-JNK, p-p38, p-c-fos, and p-c-jun were decreased by 63.6%, 64.3%, 33.4%, 59.3%, and 43.1%, respectively, after treatment with 100 µg/mL PASE.

### 2.10. Effect of PASE on Nrf2/ARE Translocation in UVB-Exposed HaCaT Cells

**Figure 7:**
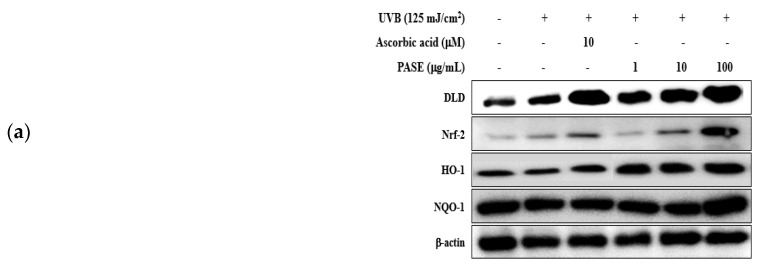

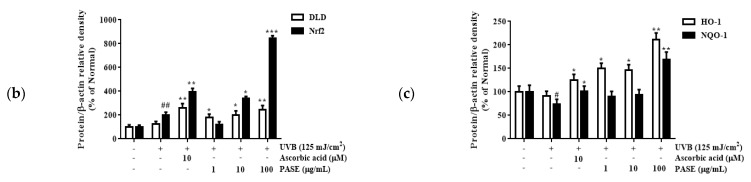
PASE stimulated the Nrf2/ARE-signaling protein levels in UVB-exposed HaCaT cells: Phosphorylated and non-phosphorylated forms of DLD, Nrf2, HO-1, and NQO-1 (**a**); the band intensities (**b**,**c**). Data are shown as means ± SDs of three independent experiments. # *p* < 0.05, ## *p* < 0.01, compared with non-exposed group, * *p* < 0.05, ** *p* < 0.01, *** *p* < 0.001 compared with only UVB-exposed group.

The main function of UV-targeted DLD in the skin might be to act as an antioxidant. Additionally, the Nrf2/ARE pathway plays an important role in the maintenance of a proper redox balance. To determine the effects of PASE treatment on this pathway, we measured DLD, Nrf2, HO-1, and NQO-1 protein levels using western blot (Figure 7). Compared with the levels found in UVB-exposed cells, a high PASE concentration (100 μg/mL) effectively increased the expression of total DLD and Nfr2 by 93.7% and 318.5%, respectively. Moreover, HO-1 and NQO-1 levels were also enhanced in cells treated with PASE. Indeed, 100 µg/mL PASE upregulated HO-1 and NQO-1 expression by 133.3% and 129.8%, respectively.

## 3. Discussion

*P angularis* L. is native to Northeast Asia and has been traditionally used for relieving edema, eliminating inflammation, and alleviating poisoning symptoms. *P. angularis* is rich in vitamin B1, which helps to recover from fatigue and is effective against symptoms, such as digestion, absorption, and memory loss. It has also been reported to exert antidiabetic, antioxidant, and anti-inflammatory effects. *P. angularis* seed coats, which are rich in polyphenols, have recently been shown to inhibit melanin biosynthesis and attenuate vascular oxidative stress in spontaneously hypertensive rats. However, no studies investigated the therapeutic effects of PASE against UVB-induced photoaging. Thus, the present study investigated the effects of PASE treatment in UVB-exposed HaCaT cells to clarify whether PASE had a therapeutic effect against UVB-induced photoaging and whether PASE can be used as an effective anti-photoaging ingredient in cosmetic products.

The increase in ROS in skin cells due to UV causes skin photo-aging and is known as a facilitating factor. Drugs derived from natural products can remove ROS and free radicals to prevent UVB-exposed photoaging while having few or no side effects [20]. It is known that excellent DPPH and ABTS radical erasing ability has the effect of reducing ROS generation by UVB. Here, we showed that PASE exhibits antioxidant properties by scavenging DPPH and ABTS radicals (Figure 2). Indeed, ROS levels decreased by 30.9%, 46.7%, and 63.2%, as PASE concentration increased (1, 10, and 100 μg/mL, respectively) (Figure 3).

The cellular antioxidant system is considered the most important endogenous defense system against oxidative stress. The Nrf2/ARE signaling pathway has been reported to be the key regulator response to oxidative stress, and a variety of natural products have been shown to promote the translocation of Nrf2 to the nucleus [21]. For example, damiana leaves extract was reported to protect against UVB-induced damage by regulating the AP-1 and Nrf2/ARE pathways in HaCaT cells [22,23]. To determine the antioxidant mechanisms activated by PASE, we analyzed the expression of antioxidant factors from the Nrf2/ARE signaling pathway in UVB-exposed HaCaT cells. Treatment of keratinocytes with 100 µg/mL PASE after UVB irradiation stimulated the expression of Nrf2 protein by 318.4% compared with that in untreated UVB-exposed cells. Moreover, HO-1 and NQO-1 levels were dramatically elevated by PASE treatment. As shown in Figure 7, 100 µg/mL PASE increased the expression of HO-1 and NQO-1 proteins by 133.3% and 129.8%. Interestingly, the effective treatment concentration was different than that used for damiana leaves extract, suggesting that PASE was more efficacious in increasing HO-1 and NQO-1 expression.

Under normal conditions, the skin generates enzymes, such as elastase and collagenase, at a similar rate as the aging process happens and age increases [24]. However, in the case of skin photoaging caused by exposure to UV over a long period of time, histological changes include irregular changes in keratinocytes, fibroblasts, and inflammatory cells to increase, thinning of blood vessel walls, and the reduction in type I and III collagen in the derm are accelerated. Therefore, collagenase and elastase inhibitors can be used as potential treatments to prevent photoaging and wrinkle formation. Here, to observe the collagenase and elastase inhibited by PASE, we verified using a spectrophotometric method. Collagenase and elastase activity were reduced by PASE treatment. Especially, 100 µg/mL PASE decreased the activation of collagenase and elastase by 37.9% and 27.7% (Figure 4a,b).

The destruction of skin collagen is mainly caused by MMPs secreted by epithelial keratinocytes and dermal fibroblasts [25]. MMPs are enzymes that degrade the extracellular matrix of the skin and are known to play an important role in inflammatory reactions, cancer metastasis, and skin aging. Thus, we focused on the targets of PASE’s action in the induction of the MMP-1 and MMP-3 after UVB irradiation. The increase in MMP-1 and MMP-3 was significantly reduced in the presence of PASE in a concentration-dependent manner (Figure 4c,d).

The MMP-1 promoter contains an AP-1 binding site. Thus, a higher AP-1 expression leads to increased production of MMP-1 [26,27]. It has been previously reported that Scutellariae Radix exerts protective effects against UVB-induced photoaging in HaCaT cells by targeting MAPK/AP-1 pathways [28,29]. Here, MAPK is an upstream regulator of AP-1, which is activated by ROS induced by UV exposure. Ascorbic acid, which is typically used as a positive control in experiments assessing antioxidant properties, has also been reported to regulate c-jun and c-fos activities by inhibiting MAPKs. We showed that 100 µg/mL PASE significantly suppressed the expression of p-ERK, p-JNK, and p-p38 by 33.3%, 63.5%, and 64.2%, respectively. These are all MAPK targets, the expression of which was increased after UVB irradiation of HaCaT cells (Figure 6a,b).

Loss of type I collagen, the main structural component of human skin, is largely responsible for visible indicators of aging. TGF-β is a major factor regulating the biosynthesis of type 1 collagen in fibroblasts as well as numerous cellular activities [30]. Smad7 prevents the interaction between Smad2/3 and TGF-β receptors, providing a promising means of reducing TGF-β1 signaling and inhibiting photoaging. Choi et al. reported that HaCaT cells recovered from UVB-induced damage by regulating the TGF-β/Smad pathways [31]. The present data were consistent with those from this study. UVB induced matrix degradation and decreased collagen production by regulating TGF-β1 and Smad7 levels. The expression of TGF-β1 mRNA and protein was 268.4% and 286.1%, respectively, higher in UVB-exposed HaCaT cells treated with 100 μg/mL PASE than that in the UVB-exposed control group (Figure 5a,c). However, PASE (100 μg/mL) treatment inhibited the expression of Smad7 proteins by 86.2% compared to that in the UVB-exposed control group. These results confirmed the PASE mechanism of action. Therefore, it is reasonable to assume that PASE recovered procollagen type I expression by activating TGF-β1 through the inhibition of Smad 7 in HaCaT cells.

## 4. Materials and Methods

### 4.1. Sample Preparations

*P. angularis* seed was purchased from Gyeongginongsan Co., Ltd. (Yeoju, Korea). A total of 100 g *P. angularis* seed powder was extracted in 500 mL of 70% ethanol by shaking on a Twist shaker for 24 h at room temperature. Then, the extract solution was filtered using 0.45-μm filter paper (Whatman, Kansas, MO, USA), concentrated in a water bath under vacuum (EYELA WORLD—Tokyo Rikakikai Co., LTD., Koishikawa, Bunkyo, Japan), frozen, and lyophilized to yield the ethanol extract (PASE). The yield was 10.44%. The voucher specimen was stored in the laboratory at Snowwhitefactory Co., Ltd. (Seoul, Korea).

### 4.2. High-Performance Liquid Chromatography (HPLC) Analysis

PASE was prepared at the concentration of 2 mg/mL in 50% methanol. Serial dilutions (2.5, 25, 125, 250, 500, and 1000 µg/mL) of the standard compound (catechin-7-O-β-D-glucopyranoside) in methanol were prepared. HPLC was performed on a Dionex Chromelon TM chromatography data system with P580 and UVD100 detectors (Thermo Fisher Scientific Inc., Waltham, MA, USA). Chromatographic separation was conducted on an Inno C-18 column (5 μm, 4.6 × 250 nm; Young Jin). The column temperature was 25 ℃, the flow rate was 1.0 mL/min, and the injected volume was 10 µL.

### 4.3. Total Phenolic and Flavonoid Contents

The total phenolic content of PASE was determined based on the Folin–Ciocalteu colorimetric method [32]. Briefly, either standard gallic acid (6.25–100 µg/mL) or PASE extract was incubated with 1 M Folin–Ciocalteu reagent, 0.7 M sodium carbonate in NaOH, and the mixture was reacted for 1 h. The absorbance values were measured at a wavelength of 625 nm. The total flavonoid content of PASE was determined based on the aluminum chloride colorimetric method [33]. Briefly, 50 mg/mL of sodium nitrate was mixed with either standard quercetin (0.03125–1 mg/mL) or PASE extract. Aluminum chloride and 1M sodium hydroxide were added and incubated. Optical density was determined at a wavelength of 450 nm. The determinations were estimated using a microplate reader (Molecular Devices FilterMax F5; San Francisco, CA, USA). The total phenol and flavonoid contents were expressed as gallic acid and quercetin equivalents, respectively, in mg per gram of PASE extract.

### 4.4. 1,1-Diphenyl-2-Picrylhydrazyl (DPPH) and 2,2′-Azino-Bis-(3-Ethylbenzothiazoline)-6-Sulfonic Acid (ABTS) Radical Scavenging Activity

The antioxidant action of PASE was investigated by DPPH (PubChem CID: 2375032) and ABTS (PubChem CID: 5464076). For the DPPH assay, aliquots of 40 µL PASE (1–250 µg/mL) were incubated in each well of a 96-well plate with 160 µL of DPPH solution in the dark at 37 °C for 30 min. The optical density was determined at a wavelength of 595 nm. An ABTS solution was performed by reacting 2.5 mM ABTS with 1 mM 2,2’-azobis(2-amidinopropane) dihydrochloride (AAPH) and 150 mM sodium chloride. In each well of a 96-well plate, 100 µL of ABTS solution and an aliquot of 100 µL PASE (1–250 µg/mL) were mixed in the dark at 37 °C for 10 min. The optical density was determined at a wavelength of 405 nm. Ascorbic acid as a positive control was used. The inhibitory effect of the sample on DPPH and ABTS radical formation was evaluated using the following formula:

$$\mathrm{DPPH}\;\mathrm{and}\;\mathrm{ABTS}\;\mathrm{radical}\;\mathrm{inhibition}\;(\%)=\frac{{(\mathrm{OD}}_{0}-{\mathrm{OD}}_{x})}{{\mathrm{OD}}_{0}}\;\times \;100$$

with OD0 being the optical density measured for the negative control and ODx, which measured for the different PASE or ascorbic acid concentrations.

### 4.5. Cell Culture and Treatment

HaCaT cells were grown in DMEM (Hyclone) supplemented with 10% heat-inactivated FBS and a 1% penicillin/streptomycin solution in 100-mm cell culture plates (Corning, Corning, NY, USA). HaCaT cells were exposed to UVB (125 mJ/cm^2^) radiation using a UVB irradiation machine by a Bio-Link BLX-312(Vilber Lourmat GmbH, Marne-la-Vallée France). Subsequently, cells were rinsed with 1× PBS, and a fresh serum-free medium containing PASE (1, 10, or 100 µg/mL) or 10 µM ascorbic acid (positive control) was added to each plate.

### 4.6. Cytotoxicity

After UVB irradiation and 24 h of treatment with PASE, an aliquot of 20 µL MTT, at the final concentration of 5 mg/mL, was added and the cells were further incubated for 4 h. Then, the medium was discarded and 800 µL of DMSO was added to solubilize formazan crystals. The optical density was measured at a wavelength of 595 nm.

### 4.7. Reactive Oxygen Species (ROS)

For ROS assay, after 24 h of sample treatment and UVB exposure, the supernatant was discarded, and the cells were incubated with 30 µM 2′7′-dichlorofluorescein diacetate (DCFH-DA) (Sigma-Aldrich, St. Louis, MO, USA) at 37 °C for 30 min. Then, the cells were washed two times with cooled 1X PBS and assembled by using 0.25% and 0.05% trypsin EDTA (Gibco). A flow cytometer system (BD Biosciences, Franklin Lakes, NJ, USA) assessed intracellular ROS quantitation. The data were analyzed by using FCS 6 plus Research Edition software.

### 4.8. Collagenase and Elastase Inhibition Assay

After 24 h of UVB irradiation and PASE treatment, an aliquot of 1 mL of cell culture medium was collected and centrifuged at 7500 rpm for 5 min. For collagenase inhibition assay, a fixed weight of 1 mg of Azo dye-impregnated collagen was measured in the test tubes, and then the homogenization proceeded after the addition of an 800 µL of 0.1 M Tris-HCl (pH 7) and a 100 µL sample was placed into each of the test tubes. A 100 µL collagenase (200 units/mL) was immediately mixed into the mixture and incubated at 43 °C for 1 h. The optical density was measured at a wavelength of 550 nm. In the elastase inhibition assay, a mixture of AAAPVN elastase substrate in 0.2 M Tris-HCl buffer solution (pH 8.0) was prepared to obtain a concentration of 1.6 mM. The elastase substrate was mixed with the 10 µL sample in the 96-well plates and preincubated at 25 °C for 10 min. After the preincubation, the reaction was initiated by adding 10 µL of elastase from the porcine pancreas (7.5 units/mL) in Tris solution buffer to the preincubated mixtures. The optical density was measured at a wavelength of 410 nm. Each sample was reverted to three times.

### 4.9. Enzyme-Linked Immunosorbent Assay (ELISA)

After 72 h of UVB irradiation and PASE treatment, an aliquot of 1 mL of cell culture medium was collected and centrifuged at 7500 rpm for 5 min. Then, 880 µL of supernatant was transferred into new tubes and stored at −20 °C until further analysis. The release of MMP-1 and MMP-3 in the medium was determined using commercially available ELISA kits following the manufacturer’s instructions. Each sample was reverted to three times.

### 4.10. Reverse Transcription-Polymerase Chain Reaction (RT-PCR) Analysis

Total cellular RNA was separated using TRIZOL reagent according to the manufacturer’s guidelines (Invitrogen Life Technologies, Carlsbad, CA, USA). RNA samples were quantified, and a total of 3 μg RNA was reverse transcribed using 200 units of reverse transcriptase and 0.5 µg/µL oligo-(dT)15 dimer (Bioneer CO., Daejeon, Korea). The cDNA was synthesized at 42 °C for 60 min and then incubated at 94 °C for 5 min to stop the reaction. Amplified products (Supplementary Table S1) were analyzed by electrophoresis on a 2.0% agarose gel and detected by nucleic acid staining (NobleBio Inc., Gyeonggi, Korea) under UV illumination. Glyceraldehyde 3-phosphate (GAPDH) was used for normalization.

### 4.11. Western Blot Analysis

HaCaT cells were harvested after UVB irradiation and lysed in a radioimmunoprecipitation assay (RIPA) buffer for at least 1 h. Then, samples were centrifuged at 12,000 rpm for 15 min to obtain total protein extracts. The protein concentration was calibrated using BCA kits (Thermo scientific, Rockford, IL, USA). Homogenized proteins were separated by sodium dodecyl sulfate-polyacrylamide gel electrophoresis (SDS-PAGE) and transferred to a polyvinylidene fluoride (PVDF) membrane. Then, membranes were blocked in 5% skim milk or 5% BSA for 1 h. After several washing steps with 1× TBST, membranes were incubated with primary antibodies overnight at 4 °C. After incubation with a secondary antibody, the protein levels were determined using chemiluminescence detection ECL reagents (Fujifilm, LAS-4000, Tokyo, Japan) and ImageMaster™ 17 2D Elite software, version 3.1 (Amersham Pharmacia Biotech, Piscataway, NJ, USA). Histone or β-actin levels were used for normalizing the amounts of either nuclear or total protein extracts, respectively.

### 4.12. Statistical Analysis

The data were analyzed using a statistical analysis system (GraphPad Prism 7). All the quantitative data are expressed as means ± SDs. The significance of differences was determined using a one-way analysis of variance (ANOVA) with the Student–Newman–Keuls test for multiple comparisons. Statistical significance was considered for *p* < 0.05. All experiments were performed independently three times.

## 5. Conclusions

In conclusion, PASE has the potential to prevent ROS formation induced by UVB exposure by targeting specific pathways. Our results demonstrate PASE inhibited MAPK/AP-1 signaling and regulated the TGF-β/Smad and Nrf2/ARE pathways in UVB-exposed HaCaT cells. Therefore, PASE might effectively protect the skin from damage caused by UVB and is potentially valuable as a new cosmetic ingredient with anti-aging and anti-wrinkle effects.

## 6. Patents

Tae-Hoo Yi et al. Composition for improving skin conditions comprising catechin glycoside and method for improving skin conditions using the same. KR Patent 10-2152932, filed on 14 January 2014 and issued on 1 September 2020.

## Data Availability

The data presented in this study are available in this paper.

## Supplementary Materials

The following supporting information can be downloaded at: https://www.mdpi.com/article/10.3390/molecules28031407/s1, Table S1: Oligonucleotide primers used for RT-PCR.
