# Supplementary material for: Anti-Photoaging Effect of Phaseolus angularis L. Extract on UVB-Exposed HaCaT Keratinocytes and Possibilities as Cosmetic Materials
Source: Molecules. 2023 Feb 1;28(3):1407. doi: 10.3390/molecules28031407 (PMC9919029; doi:10.3390/molecules28031407)
Supplement: Supplementary file 1 [file molecules-28-01407-s001.zip › molecules-2145354-supplementary.pdf]

Table S1. Oligonucleotide primers used for RT-PCR

NCBI accession code	Primer[a]		Sequence (5'-3')
XM_011526432.2	Human GAPDH	Sense	ACC ACA GTC CAT GCC ATC AC
		Antisense	CCA CCA CCC TGT TGC TGT AG
NM_001145938.2	Human MMP-1	Sense	TGC GCA CAA ATC CCT TCT AC
		Antisense	TTC AAG CCC ATT TGG CAG TT
NM_000088.4	Human Procollagen Type I	Sense	CTC GAG GTG GAC ACC CT
		Antisense	CAG CTG GAT GGC CAC ATC GG
NG_013364.1	Human TGF-β1	Sense	GCC CTG GAC ACC AAC TAT TGC
		Antisense	GCT GCA CTT GCA GGA GCG CAC

a: Primer design based on the NCBI/Primer-BLAST tool with standard parameters
